# Age-stratified prevalence of colorectal adenoma and advanced neoplasia in adults aged 18–39 years: a single-center cross-sectional study of 2, 149 colonoscopies

**DOI:** 10.3389/fonc.2026.1897209

**Published:** 2026-09-14

**Authors:** Yiming Ding, Jiani Liu, Huihui Li, Xiangchun Lin

**Affiliations:** Department of Gastroenterology, Peking University International Hospital, Beijing, China

**Keywords:** adenoma, colorectal neoplasia, metabolic syndrome, serrated polyps, young adult

## Abstract

**Objective:**

This study aims to investigate the pathogenic characteristics of colorectal polyps and the influence of metabolic factors among individuals aged 18 to 39 years.

**Methods:**

A retrospective analysis was performed on the clinical and pathological data of 2,149 young adults who underwent colonoscopy, and a comparative analysis was conducted by age group.

**Results:**

The adenoma detection rate increased significantly with age (4.58% → 13.23%, *p* < 0.01), while the detection rate of serrated polyps remained stable between 7.44% and 12.89%. The detection rate of advanced neoplasia also showed an upward trend (1.26% → 3.26%). The detection rate of advanced neoplasia in men aged 35 to 39 years reached 4.89%, which was 3.4 times that of women in the same age group. No statistically significant difference was observed in the clinical characteristics between adenomas and serrated polyps. However, for serrated polyps, each 1 kg/m^2^ increase in BMI was associated with a 13% increase in the risk of left colon lesions (OR = 1.13, *p* = 0.036), and this association was not observed in adenomas.

**Conclusions:**

Men aged 35 to 39 years constitute a high-risk subgroup for advanced colorectal neoplasia. The effect of metabolic factors on the location of colorectal polyps exhibits pathological type heterogeneity.

## Introduction

Colorectal cancer is the third most common malignant tumor globally and the second leading cause of cancer-related mortality (1). In 2022, approximately 4,824,703 new cancer cases and 2,574,176 cancer-related deaths were recorded in China, contributing to 71,037,170 disability-adjusted life years (DALYs) (2). Over the past two decades, while the incidence of colorectal cancer among people aged 50 and above has continued to decline in most developed countries, the incidence of early-onset colorectal cancer (EO-CRC; generally defined as colorectal cancer diagnosed before the age of 50) has increased substantially since the 1990s, especially in high-income regions, and has become a global public health challenge (3–5). Projections indicate that by 2030, EO-CRC will account for 11% of all global colorectal cancer cases (6).

Patients with EO-CRC frequently experience diagnostic delay. Research indicates that the median interval from the onset of initial symptoms to definitive diagnosis ranges from approximately 4 to 6 months, which is significantly longer than that of middle-aged and elderly patients (7, 8). The main contributing factors include insufficient attention to symptoms among young patients, low vigilance for malignant tumors among first-visit physicians, and the lack of systematic screening coverage for young populations. Consequently, a higher proportion of patients with EO-CRC are diagnosed at a locally advanced stage or with distant metastasis, leading to poorer prognosis. Multiple national guidelines have lowered the starting age of colonoscopy screening for average-risk populations to 40–45 years, which highlights the necessity of early intervention for young high-risk populations (9, 10).

However, systematic reports on the detection of colorectal polyps among the young population aged 18 to 39 years remain limited at present (11, 12). This age group is generally considered to be at low risk for colorectal cancer; however, whether the risk varies across age subgroups within this range remains poorly characterized. Furthermore, with the increasing prevalence of metabolic syndrome and obesity among young people, the association between these conditions and colorectal cancer in this specific age group warrants further investigation.

As the primary precancerous lesion, the detection rate of colorectal adenoma is a core indicator for evaluating the quality of colonoscopy and the risk of colorectal cancer (13). Serrated polyps represent another key carcinogenic pathway, accounting for approximately 15% to 30% of all colorectal cancers (14). Among the young population, the distribution characteristics, similarities and differences in risk factors, and age-dependent trends of adenomas and serrated polyps remain unclear, which limits the accurate assessment of colorectal cancer risk in this population.

This study focused on a cohort of adults aged 18 to 39 years undergoing colonoscopy to map the changing landscape of colorectal polyps across this age spectrum. By comparing the metabolic characteristics of adenomatous and serrated pathways, we sought to identify specific risk factors that could inform targeted screening strategies for young adults.

## Methods

### Research design and ethics approval

This retrospective cross-sectional study was approved by the Institutional Review Board (IRB) of a university-affiliated tertiary hospital in Beijing, China (Approval No. 2025-KY-0118-01). As no additional interventions were administered, the study utilized de-identified routine clinical data. The IRB granted a waiver of informed consent in accordance with national regulations regarding the ethical review of biomedical research.

### Research objects

We retrospectively reviewed data from patients aged 18–39 years who underwent colonoscopy at a university-affiliated tertiary hospital in Beijing, China, between 1 January 2023 and 31 December 2024. The inclusion criteria were as follows: (1) age 18–39 years and (2) complete clinical and colonoscopy data. The exclusion criteria were as follows: (1) history of inflammatory bowel disease, familial adenomatous polyposis, or colorectal cancer; (2) previous colorectal surgery; and (3) inadequate bowel preparation precluding adequate mucosal visualization.

### Terminology

In this study, “colorectal polyps” is used as a generic term for any endoscopically detected and pathologically confirmed polypoid lesion, encompassing both nonneoplastic and neoplastic polyps. Polyp size was described using standard endoscopic terminology, with “diminutive” defined as ≤ 5 mm and “small” as < 10 mm. “Colorectal neoplasia” specifically refers to lesions with established malignant potential. In accordance with the 2019 WHO Classification of Tumors of the Digestive System (15), colorectal epithelial precursor lesions comprise two broad categories: conventional adenomas (tubular, tubulovillous, or villous) and serrated polyps. Serrated polyps comprise three subtypes: hyperplastic polyps (HPs), sessile serrated lesions (SSLs; with or without dysplasia), and traditional serrated adenomas (TSAs) (15). Although most HPs, particularly small (< 10 mm) lesions in the rectosigmoid, carry a very low risk of progression, HPs ≥ 10 mm, all SSLs, and all TSAs have demonstrable malignant potential and warrant heightened endoscopic surveillance; current guidelines recommend repeat colonoscopy within 3–5 years for such lesions (16, 17). To preserve consistency with the WHO taxonomic framework, all serrated polyps, including small HPs, were classified within the serrated polyp category in the present analysis, whereas the higher-risk subgroup was analyzed separately as advanced serrated polyps. Advanced neoplasia comprises advanced adenoma (diameter ≥ 10 mm, or with high-grade intraepithelial neoplasia, or with villous components) and advanced serrated polyp (a serrated polyp with a diameter ≥ 10 mm or with dysplasia. Advanced neoplasia comprises advanced adenoma and advanced serrated polyp.

### Data collection and indicator definition

Demographic information, clinical characteristics (including colonoscopy indications and comorbidities), laboratory examination results, and polyp pathological features (including location, size, quantity, and pathological type) of patients were collected via the hospital electronic medical record system and digestive endoscopy information system. Fatty liver was defined based on abdominal imaging (ultrasonography, CT, or MRI) performed within six months before the colonoscopy that indicated hepatic steatosis. All histopathological diagnoses were rendered by the gastrointestinal pathology team at our institution (Peking University International Hospital, a tertiary university-affiliated hospital) in accordance with the 2019 WHO Classification of Tumors of the Digestive System. The primary outcome indicators are defined as follows: polyp detection rate refers to the proportion of examinees with at least one polyp of any pathological type detected; adenoma detection rate is the proportion of examinees with at least one conventional adenoma detected; serrated polyps detection rate is the proportion of examinees with at least one serrated polyp detected; advanced adenoma detection rate is the proportion of examinees with at least one advanced adenoma detected; advanced serrated polyp detection rate is the proportion of examinees with at least one advanced serrated polyp (diameter ≥ 10 mm or with dysplasia) detected (18); and advanced neoplasia detection rate is the proportion of examinees with at least one advanced adenoma or at least one advanced serrated polyp detected, and this indicator is the composite endpoint of the two indicators.

### Sample size and enrollment

The sample size was calculated based on the adenoma detection rates observed in our institution’s prior data: approximately 4% for the 18–29-year group, 8% for the 30–34-year group, and 13% for the 35–39-year group. With a two-sided α of 0.05 and a power of 80%, the noncentrality parameter method for the Cochran–Armitage trend test estimated that a minimum of 426 participants (containing approximately 33 adenoma cases) would be required across the three groups. The present study enrolled 2,149 participants with 177 adenomas, exceeding this requirement. Ultimately, we consecutively enrolled 2,149 eligible participants (18–29 years: *n* = 874; 30–34 years: *n* = 693; 35–39 years: *n* = 582), among whom 412 polyps were identified. Of the 412 participants with polyps, 49 were found to have only nonneoplastic lesions (inflammatory polyps, polypoid mucosal chronic inflammation) and were excluded from the pathological subgroup analysis, which focused on conventional adenomas and serrated polyps.

### Statistical analysis

Continuous variables are expressed as mean ± standard deviation, while categorical variables are presented as frequency (percentage). Comparisons between groups were performed using one-way analysis of variance, independent samples *t*-test, Chi-square test, or Fisher’s exact test, and trend analysis was conducted using the Cochran–Armitage test. Multinomial logistic regression was used to analyze the age × gender interaction effect and the association between metabolic factors and polyp location, and the significance of interaction terms was evaluated by the likelihood ratio test. The significance level was set at *α* = 0.05 (two-tailed). All statistical analyses were performed using Python 3.12.

## Results

A total of 2,149 young participants aged 18–39 years who underwent colonoscopy were enrolled in this study, among whom 412 were diagnosed with polyps. The participants were divided into three groups by age: the 18–29-year group (*n* = 112), the 30–34-year group (*n* = 150), and the 35–39-year group (*n* = 150). The baseline characteristics of the three groups are shown in **Table 1**. No statistically significant differences were observed in gender composition and family history of colorectal cancer among the three groups. Metabolism-related indicators showed a consistent upward trend with increasing age: BMI increased from 24.3 to 25.4 kg/m^2^, the prevalence of fatty liver rose from 13.4% to 22.0%, and the prevalence of hypertension reached 10.0% in the 35–39-year group. The proportions of smoking history and drinking history also tended to increase with age. Regarding blood lipid profiles, the levels of low-density lipoprotein cholesterol (LDL-C), total cholesterol, and triglycerides in the 35–39-year group were higher than those in the other two groups. The distribution of colonoscopy indications varied significantly with age: the proportion of examinations for health screening increased from 4.5% to 30.7%, while the proportion of examinations for hematochezia/melena decreased from 14.3% to 4.0%. The distribution of polyps in the left and right hemicolon was generally balanced, and no significant side predominance was observed among the three groups. No statistically significant differences were found in the levels of alanine aminotransferase (ALT), aspartate aminotransferase (AST), gamma-glutamyl transferase (GGT), uric acid, and fasting blood glucose among the three groups.

**Table 1:** Baseline characteristics of patients with colorectal polyps, stratified by age group.

Category	Variable	18–29 years (*n* = 112)	30–34 years (*n* = 150)	35–39 years (*n* = 150)
Demographics	Age (years, mean ± SD)	27.5 ± 2.1	33.1 ± 1.5	37.7 ± 1.4
	Gender (men, %)	70 (62.5%)	91 (60.7%)	94 (62.7%)
	BMI (kg/m^2^, mean ± SD)	24.3 ± 4.8	24.8 ± 4.1	25.4 ± 4.1
Clinical history	Inpatient source (%)	10 (8.9%)	16 (10.7%)	22 (14.7%)
	Painless anesthesia (%)	95 (84.8%)	142 (94.7%)	140 (93.3%)
	Family history (%)	6 (5.4%)	8 (5.3%)	6 (4.0%)
Endoscopy indications	Health check-up (%)	5 (4.5%)	31 (20.7%)	46 (30.7%)
	Abdominal pain (%)	18 (16.1%)	8 (5.3%)	8 (5.3%)
	Bloating (%)	12 (10.7%)	15 (10.0%)	10 (6.7%)
	Bowel habit change (%)	61 (54.5%)	72 (48.0%)	74 (49.3%)
	Hematochezia/melena (%)	16 (14.3%)	18 (12.0%)	6 (4.0%)
	Other indications (%)	0 (0.0%)	6 (4.0%)	6 (4.0%)
Polyp location	Left only (%)	56 (50.0%)	73 (48.7%)	67 (44.7%)
	Right only (%)	42(37.5%)	62 (41.3%)	58 (38.7%)
	Bilateral (%)	14 (12.5%)	15 (10.0%)	25 (16.7%)
Lifestyle and comorbidities	Smoking history (%)	1 (0.9%)	8 (5.3%)	8 (5.3%)
	Alcohol history (%)	3 (2.7%)	9 (6.0%)	12 (8.0%)
	Hypertension (HTN, %)	3 (2.7%)	4 (2.7%)	15 (10.0%)
	Diabetes (DM, %)	1 (0.9%)	1 (0.7%)	4 (2.7%)
	Fatty liver (NAFLD, %)	15 (13.4%)	27 (18.0%)	33 (22.0%)
Laboratory tests	ALT (U/L)	26.9 ± 28.4	26.4 ± 20.2	26.3 ± 17.1
	AST (U/L)	24.4 ± 15.5	23.5 ± 7.9	23.4 ± 9.1
	GGT (U/L)	22.8 ± 17.0	27.6 ± 31.9	29.6 ± 23.6
	LDL (mmol/L)	2.8 ± 0.7	2.8 ± 0.6	3.0 ± 0.6
	Total cholesterol (mmol/L)	4.5 ± 0.7	4.5 ± 0.7	4.7 ± 0.7
	Triglycerides (mmol/L)	1.2 ± 0.9	1.3 ± 1.0	1.4 ± 1.0
	Uric acid (μmol/L)	418.2 ± 98.8	400.7 ± 104.5	399.9 ± 113.3
	Fasting glucose (mmol/L)	5.4 ± 0.9	5.4 ± 0.6	5.4 ± 0.8

All patients with conventional adenomas and/or serrated polyps were classified into three mutually exclusive pathological groups: adenoma only (*n* = 136), serrated polyps only (*n* = 186), and mixed lesion (coexisting conventional adenoma and serrated polyp, *n* = 41). The 227 serrated-polyp cases comprised hyperplastic polyp (*n* = 190), SSL without dysplasia (*n* = 43), SSL with dysplasia (*n* = 3), and traditional serrated adenoma (*n* = 4); as some patients harbored more than one subtype, these subtype counts are nonexclusive and exceed 227. Baseline characteristics were compared across the three groups (**Table 2**). Age, gender, BMI, family history of colorectal cancer, most endoscopy indications, and all laboratory indicators did not differ significantly among the three groups (all *p* > 0.05). In contrast, inpatient source (*p* < 0.001), smoking history (*p* = 0.034), alcohol history (*p* = 0.017), and polyp location differed significantly across the groups, with bilateral involvement far more frequent in the mixed-lesion group than in the adenoma-only or serrated-only groups (48.8% vs. 8.8% and 11.3%; *p* < 0.001). The prevalence of fatty liver did not differ significantly among the three groups (20.6%, 17.2%, and 17.1%; *p* = 0.720).

**Table 2:** Baseline characteristics of young adults with colorectal polyps, stratified by histopathology.

Category	Variable	Adenoma only (*n* = 136)	Serrated only (*n* = 186)	Mixed (*n* = 41)	*p*-value
Demographics	Age (years, mean ± SD)	33.4 ± 4.4	32.9 ± 4.4	34.0 ± 4.0	0.281
	Gender (men, %)	87 (64.0%)	107 (57.5%)	28 (68.3%)	0.307
	BMI (kg/m^2^, mean ± SD)	25.4 ± 4.6	24.6 ± 4.4	24.8 ± 4.0	0.183
Clinical history	Inpatient source (%)	16(11.8%)	16(8.6%)	14(34.1%)	< 0.001
	Painless anesthesia (%)	123 (90.4%)	172 (92.5%)	36 (87.8%)	0.589
	Family history of CRC/polyp (%)	9 (6.6%)	6 (3.2%)	4 (9.8%)	0.155
Endoscopy indications	Health check-up (%)	28 (20.6%)	36 (19.4%)	9 (22.0%)	0.918
	Abdominal pain (%)	12 (8.8%)	16 (8.6%)	2 (4.9%)	0.703
	Bloating (%)	11 (8.1%)	19 (10.2%)	3 (7.3%)	0.739
	Bowel habit change (%)	71 (52.2%)	91 (48.9%)	20 (48.8%)	0.830
	Hematochezia/melena (%)	9 (6.6%)	20 (10.8%)	4 (9.8%)	0.438
Polyp location	Left only (%)	72 (52.9%)	85 (45.7%)	13 (31.7%)	0.052
	Right only (%)	52 (38.2%)	80 (43.0%)	8 (19.5%)	0.020
	Bilateral (%)	12 (8.8%)	21 (11.3%)	20 (48.8%)	< 0.001
Lifestyle and comorbidities	Smoking history (%)	4 (2.9%)	7 (3.8%)	5 (12.2%)	0.034
	Alcohol history (%)	7 (5.1%)	10 (5.4%)	7 (17.1%)	0.017
	Hypertension (%)	7 (5.1%)	9 (4.8%)	4 (9.8%)	0.446
	Diabetes (%)	3 (2.2%)	3 (1.6%)	0 (0.0%)	0.623
	Fatty liver/NAFLD (%)	28 (20.6%)	32 (17.2%)	7 (17.1%)	0.720
Laboratory tests	ALT (U/L, mean ± SD)	27.1 ± 19.7	27.1 ± 26.0	21.9 ± 11.0	0.206
	AST (U/L, mean ± SD)	23.5 ± 8.5	24.4 ± 13.8	21.7 ± 6.4	0.573
	GGT (U/L, mean ± SD)	26.3 ± 20.2	27.5 ± 29.2	24.2 ± 16.2	0.559
	LDL-C (mmol/L, mean ± SD)	3.0 ± 0.6	2.8 ± 0.7	3.0 ± 0.6	0.133
	Total cholesterol (mmol/L, mean ± SD)	4.7 ± 0.7	4.5 ± 0.7	4.6 ± 0.5	0.121
	Triglycerides (mmol/L, mean ± SD)	1.3 ± 1.1	1.3 ± 0.9	1.2 ± 0.6	0.796
	Uric acid (μmol/L, mean ± SD)	414.2 ± 105.6	398.5 ± 100.4	436.9 ± 128.1	0.067
	Fasting glucose (mmol/L, mean ± SD)	5.5 ± 0.9	5.4 ± 0.8	5.2 ± 0.4	0.072
	Eosinophils (%, mean ± SD)	2.7 ± 2.1	3.0 ± 2.3	2.5 ± 1.6	0.506

Patients were classified into three mutually exclusive groups according to pathology—adenoma only (*n* = 136), serrated only (*n* = 186), and mixed lesion (coexisting conventional adenoma and serrated polyps in the same patient, *n* = 41). Continuous variables are presented as mean ± SD (Kruskal–Wallis test); categorical variables are presented as *n* (%) (Chi-square/Fisher test).

Regarding the morphological characteristics of polyps, the proportion of solitary polyps exhibited a downward trend with increasing age (77.3% in the 18–29-year age group, 72.7% in the 30–34-year age group, and 65.3% in the 35–39-year age group; *p* = 0.033 for the trend test). The detection proportions of large polyps (diameter ≥ 10 mm) in the three groups were 7.3%, 4.0%, and 8.0%, respectively, while the proportions of multiple polyps (≥ 3 lesions) were 10.0%, 9.3%, and 14.0%, respectively. No significant age-related trend was observed for either indicator (all *p* > 0.05 for the trend test).

Among the 2,149 young participants aged 18–39 years who underwent colonoscopy in the entire cohort, the prevalence of colorectal polyps stratified by different age groups and gender is shown in **Table 3**. The overall prevalence of adenoma was 4.58% (95% confidence interval [CI]: 3.38%–6.16%) in the 18–29-year group, 8.66% (95% CI: 6.79%–10.97%) in the 30–34-year group, and 13.23% (95% CI: 10.71%–16.23%) in the 35–39 year group, showing a marked increasing trend. The overall prevalence of serrated polyps in the three groups was 7.44%, 12.55%, and 12.89%, respectively. The overall prevalence of advanced neoplasia also increased with age: 1.26% (95% CI: 0.70%–2.24%) in the 18–29-year group, 1.73% (95% CI: 1.00%–2.99%) in the 30–34-year group, and 3.26% (95% CI: 2.11%–5.03%) in the 35–39-year group. Gender-stratified analysis showed that the prevalence of adenoma was higher in men than in women across all age groups. The prevalence of advanced neoplasia in men reached 4.89% (95% CI: 2.98%–7.89%) in the 35–39-year group, which was significantly higher than that in women of the same age group (1.45%, 95% CI: 0.57%–3.66%).

**Table 3:** Age- and gender-specific prevalence of colorectal polyps in young adults aged 18–39 years undergoing colonoscopy.

Age group (years)	Gender	Total (*N*)	Adenoma (*n*, %; 95%CI)	Serrated polyps (*n*, %; 95% CI)	AN (*n*, %; 95% CI)
18–29	Both	874	40 (4.58; 3.38–6.16)	65 (7.44; 5.89–9.35)	11 (1.26; 0.70–2.24)
	Men	428	22 (5.14; 3.41–7.66)	38 (8.88; 6.54–11.93)	7 (1.64; 0.80–3.32)
	Women	446	18 (4.04; 2.58–6.28)	27 (6.05; 4.20–8.65)	4 (0.90; 0.35–2.28)
30–34	Both	693	60 (8.66; 6.79–10.97)	87 (12.55; 10.29–15.24)	12 (1.73; 1.00–2.99)
	Men	339	38 (11.21; 8.26–14.99)	52 (15.34; 11.91–19.53)	5 (1.47; 0.63–3.39)
	Women	354	22 (6.21; 4.15–9.23)	35 (9.89; 7.20–13.44)	7 (1.98; 0.96–4.01)
35–39	Both	582	77 (13.23; 10.71–16.23)	75 (12.89; 10.41–15.86)	19 (3.26; 2.11–5.03)
	Men	307	55 (17.92; 14.00–22.61)	44 (14.33; 10.85–18.69)	15 (4.89; 2.99–7.89)
	Women	275	22 (8.00; 5.34–11.83)	31 (11.27; 8.06–15.57)	4 (1.45; 0.57–3.66)

*AN*, advanced neoplasia: adenoma ≥ 10 mm, or with villous/tubulovillous histology, or with high-grade dysplasia, or serrated polyps ≥ 10 mm.

**Figure 1** illustrates the trend in the detection rates of colorectal polyps with increasing age among the population aged 18 to 39 years. The study population was divided into four subgroups by 5-year age intervals: 179 cases in the 18–24-year group, 695 cases in the 25–29-year group, 693 cases in the 30–34-year group, and 582 cases in the 35–39-year group. The results showed that the detection rates of both adenoma and serrated polyps increased in a stepwise manner with age: the detection rate of adenoma rose from 3.35% in the 18–24-year group to 13.2% in the 35–39-year group, while that of serrated polyps increased from 5.59% to 13.2%. Although the overall detection rate of advanced neoplasia was relatively low, it also exhibited the same upward trend, increasing from 1.11% to 3.26%. The Cochran–Armitage trend test confirmed that the increases in the detection rates of adenoma (*p* < 0.0001), serrated polyps (*p* < 0.0001), and advanced neoplasia (*p* = 0.0001) were all statistically significant.

**Figure 1:**
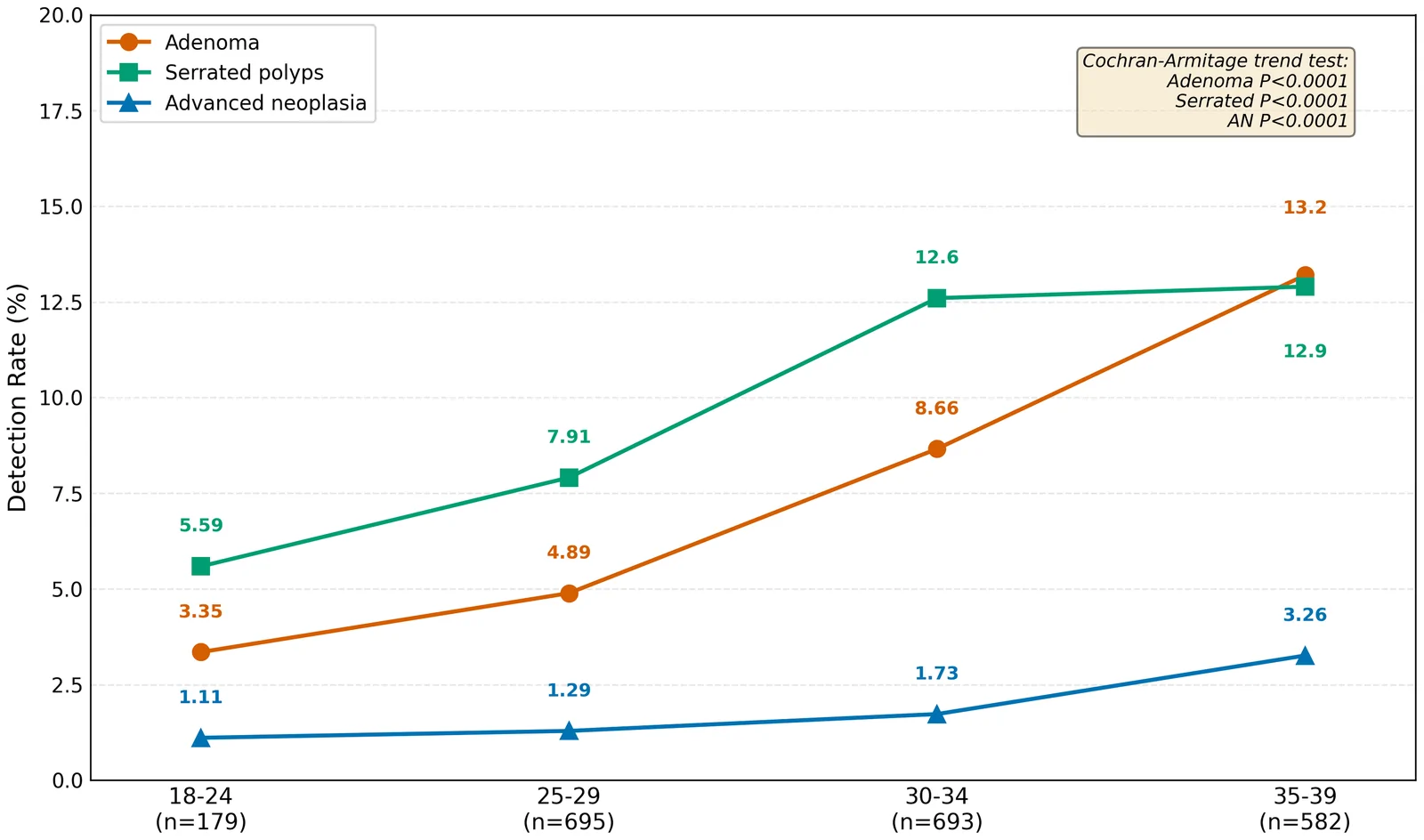
Trends in detection rates of adenoma, serrated polyps, and advanced neoplasia across age groups in a young Chinese population: a cross-sectional study.

A logistic regression model was constructed with age (continuous variable, unit: year) and gender as independent variables, and adenoma detection (yes/no) as the dependent variable (**Table 4**). After adding the age-by-gender interaction term to the main effect model, the interaction term yielded β = 0.073 (SE = 0.038), odds ratio (OR) = 1.08 (95% CI: 1.00–1.16), and *p* = 0.057. The results of the likelihood ratio test showed that the difference between model 1 (containing only main effects) and model 2 (including the interaction term) did not reach statistical significance (*χ*^2^ = 3.58, *df* = 1, *p* = 0.059). The risk of adenoma detection increased significantly with age in men, with an OR = 1.13 per 1-year increment in age (95% CI: 1.08–1.19, *p* < 0.001). In women, the risk of adenoma detection also increased with age, but the increment was smaller and did not reach statistical significance, with an OR = 1.05 per 1-year increment in age (95% CI: 0.99–1.12, *p* = 0.085).

**Table 4:** Logistic regression analysis of the age × gender interaction on adenoma detection and gender-stratified age effects.

Variable	β	SE	OR	95% CI	*p*-value
Model with interaction term					
Age (year)	0.051	0.030	1.05	0.99–1.12	0.085
Male sex	− 1.927	1.288	0.15	0.01–1.78	0.135
Age × male	0.073	0.038	1.08	1.00–1.16	0.057
Gender-stratified age effects					
Women: OR per year	–	–	1.05	0.99–1.12	0.085
Men: OR per year	–	–	1.13	1.08–1.19	< 0.001

Likelihood ratio test for age × sex interaction: *χ*^2^ = 3.58, *df* = 1, *p* = 0.059. The interaction model included age (continuous, years), gender, and the gender × sex interaction term. The reference category for gender-stratified analyses was female sex. Gender-stratified ORs per year were derived from separate logistic regression models fitted within each gender stratum.

*OR*, odds ratio; *CI*, confidence interval; *SE*, standard error.

A total of 412 patients with colorectal polyps were stratified according to pathological type and polyp location. After excluding patients with bilateral lesions and mixed pathology, 114 cases of pure adenoma were identified (65 in the left colon, 49 in the right colon), and 156 cases of pure serrated polyps were identified (84 in the left colon, 72 in the right colon) (**Table 5**). Among patients with adenoma, no statistically significant differences were observed in body mass index (BMI), fasting blood glucose, triglyceride, LDL-C, and ALT levels between left- and right-colon adenomas. Since there were only 49 cases of right-colon adenoma (events per variable [EPV] = 7), which failed to meet the minimum events-per-variable requirement for multivariate regression, only univariate comparisons were performed. Among patients with serrated polyps, the BMI of patients with left-colon serrated polyps was significantly higher than that of those with right-colon serrated polyps (*p* = 0.004). No statistically significant differences were found in fasting blood glucose, triglycerides, LDL-C, and ALT between the two groups (all *p* > 0.05). Multivariate logistic regression analysis showed that for every 1 kg/m^2^ increase in BMI, the odds of serrated polyps being located in the left colon increased by 13% (OR = 1.13, 95% CI: 1.01–1.27, *p* = 0.036). The risk of left-colon serrated polyps in men was 2.24 times that in women, but this difference did not reach a statistically significant level (95% CI: 0.98–5.13, *p* = 0.057). Age, fasting blood glucose, triglycerides, LDL-C, ALT, and hypertension were not identified as independent predictive factors (all *p* > 0.05).

**Table 5:** Metabolic characteristics of left- versus right-sided colorectal polyps, stratified by pathology.

Variable	Adenoma			Serrated		
	Left (*n* = 65)	Right (*n* = 49)	*p*-value	Left (*n* = 84)	Right (*n* = 72)	*p*-value
Age (years)	33.0 ± 4.5	33.9 ± 4.3	0.291	32.5 ± 4.2	32.8 ± 4.4	0.742
Men (*n*, %)	40 (61.5)	32 (65.3)	0.677	46 (54.8)	31 (43.1)	0.144
BMI (kg/m^2^)	24.5 ± 4.6	26.2 ± 4.4	0.053	25.6 ± 4.1	23.6 ± 4.6	0.004
FPG (mmol/L)	5.4 ± 0.9	5.6 ± 0.7	0.224	5.5 ± 0.7	5.5 ± 0.8	0.700
TG (mmol/L)	1.3 ± 0.9	1.3 ± 1.1	0.976	1.3 ± 0.9	1.3 ± 0.9	0.833
LDL-C (mmol/L)	2.9 ± 0.6	3.0 ± 0.6	0.929	2.8 ± 0.7	2.8 ± 0.7	0.668
ALT (U/L)	28.5 ± 19.0	25.2 ± 13.1	0.385	28.1 ± 22.5	27.6 ± 19.8	0.923
Multivariable analysis^a^ (OR, 95% CI)						
Age (year)				0.98 (0.90–1.08)		0.710
Male sex				2.24 (0.98–5.13)		0.057
BMI (kg/m^2^)				1.13 (1.01–1.27)		0.036
FPG (mmol/L)				1.26 (0.59–2.67)		0.549
TG (mmol/L)				0.77 (0.50–1.19)		0.244
LDL-C (per mmol/L)				1.03 (0.56–1.89)		0.921
Hypertension				0.71 (0.11–4.56)		0.720

*BMI*, body mass index; *FPG*, fasting plasma glucose; *TG*, triglycerides; *LDL-C*, low-density lipoprotein cholesterol; *ALT*, alanine aminotransferase; *OR*, odds ratio; *CI*, confidence interval.

a Multivariable logistic regression was performed only for serrated polyps. The adenoma subgroup (right-sided *n* = 49) did not meet the minimum events-per-variable criterion (EPV ≈ 7) and was therefore limited to univariate comparisons. For the serrated model, all seven listed variables were entered simultaneously; EPV = 72/7 ≈ 10.

## Discussion

In this study, a significant upward trend in the detection rates of colorectal adenomas and serrated polyps with increasing age was observed among young adults aged 18 to 39 years, which is consistent with the epidemiological characteristics of middle-aged and elderly populations, confirming that age is a core driving factor for polyp incidence in this population (19). Notably, the detection rate of serrated polyps in the 18–24-year age group (5.59%) was already close to that of adenomas, indicating that this type of lesion already has a relatively high occurrence risk in youth and cannot be neglected (20).

Our overall serrated-polyp detection rate (10.6%) exceeds the 1%–5.4% reported in earlier Chinese series (21, 22), largely because the 2019 WHO classification lowered the histologic threshold for sessile serrated lesions and our enrollment was confined entirely to 2023–2024 under high-definition colonoscopy; the predominantly symptomatic composition of our cohort (screening examinations < 30%) further contributes to this difference relative to older average-risk screening studies. When compared with the most recent Asian studies applying comparable 2019 WHO criteria, however, our rate is concordant rather than anomalous: in a large Taiwanese series of 4,712 individuals aged < 50 years (20), Yeh et al. reported a clinically significant serrated polyp (CSSP) prevalence of 10.3% and an SSL prevalence of 7.2%, while in a two-center mainland Chinese cohort of young adults aged 18–44 years, Shen et al. similarly found hyperplastic polyps to be the second most frequent polyp subtype after tubular adenomas and predominantly distal (sigmoid: 29.4%, rectum: 25.4%) (12). The convergence of these contemporary studies on a serrated detection rate of ~10%, far above the historical 1%–5.4%, indicates that the earlier “low” rates chiefly reflected under-recognition of SSLs and the restrictive definitions then in use, rather than a true scarcity of lesions.

With respect to risk factors, Yeh et al. found that SSL detection among younger individuals was driven principally by endoscopic quality—longer withdrawal time (OR = 1.17/min) and high endoscopist performance (OR = 3.35)—with age 40–49 years being an additional predictor, whereas metabolic factors (obesity, metabolic syndrome, and diabetes) that associated in univariable analysis did not retain independent significance after adjustment. Our finding that BMI was independently associated with left-sided localization of serrated polyps (OR = 1.13 kg/m^2^), without a parallel effect on adenomas, suggests a histology-specific metabolic susceptibility. Both studies concur on a male predominance among younger individuals with serrated lesions and, importantly, on a comparatively flat age trajectory of serrated/SSL detection in contrast to the steep age-related rise of adenomas.

Specifically, the detection rate of adenomas increased from 4.58% in the 18–29-year age group to 13.23% in the 35–39-year age group, and the detection rate of advanced neoplasia also rose from 1.26% to 3.26%. Although the detection rate of advanced neoplasia reached as high as 4.89% in men aged 35–39, extrapolation of these data should be conducted with caution due to the low proportion of asymptomatic individuals undergoing physical examination in this study. Comparison with international data further confirms the complexity of population composition: the overall detection rates of adenomas and high-risk adenomas in this study are both significantly lower than those in the same-age screening population in the USA (for example, 10.7% vs. 17.6% in the 30–39 age group). This is mainly attributed to the strict exclusion of individuals with definite high-risk factors such as previous polyp history and inflammatory bowel disease, resulting in a study population being mainly composed of a “low-risk symptomatic population”, which dilutes the overall positive rate. However, this “dilution effect” is reversed in the male subgroup aged 35–39: the detection rate of high-risk adenomas in this subgroup (4.89%) is comparable to that of the 40–44-year screening population in the USA (4.6%).

The detection rates of adenomas and advanced neoplasia were higher in men than in women across all age groups, and this gender difference became increasingly pronounced with age. Particularly in the 35–39-year age group, the detection rate of advanced neoplasia in men reached 4.89%, which was 3.4 times that of women in the same age group (1.45%). The marked male predominance in colorectal neoplasia observed in this study cannot be solely attributed to anatomical or physiological disparities. Recent evidence suggests that sex hormones play a pivotal role: estrogen is believed to confer a protective effect by modulating a favorable gut microbiota composition, thereby suppressing chronic inflammation and tumorigenesis, whereas androgen exposure may exert direct protumorigenic effects by increasing the abundance of opportunistic pathogens. This leads to a distinct “male-biased gut microbiome” characterized by dysbiosis, which exacerbates intestinal barrier dysfunction and drives metabolic disturbances (e.g., glycerophospholipid metabolism) that fuel cancer progression. Consequently, the higher prevalence of advanced neoplasia and the significantly increased risk of colorectal cancer mortality in men (with hazards nearly doubling for adenomas and quadrupling for high-risk polyps compared with those in women) likely stem from this synergistic effect of metabolic aberrations, unhealthy behaviors, and a biologically aggressive, male-specific tumor microenvironment (23–25).

Data from this study show that the prevalence of conditions including nonalcoholic fatty liver disease (NAFLD), hypertension, and diabetes in men aged 35–39 years is significantly higher than that in the 18–29-year age group, accompanied by a substantial increase in the proportions of smoking and alcohol consumption. The data also indicate that among individuals aged 18–39 years, metabolism-related indicators including BMI and blood lipid levels show an upward trend with increasing age, which is consistent with the increasing trend in the adenoma detection rate. This covariation suggests that components of metabolic syndrome may be associated with the risk of colorectal polyps in this age group. In fact, multiple previous studies have confirmed in different populations that NAFLD and related metabolic disorders are not only risk factors for colorectal polyps but are also important driving factors for the occurrence of colorectal cancer (26). As a visceral manifestation of insulin resistance, NAFLD is involved in the oncogenesis of colorectal cancer through mechanisms such as chronic low-grade inflammation, oxidative stress, and intestinal dysbiosis (27, 28).

In this study, the distributions of age, gender, BMI, family history, smoking and drinking history, and all laboratory indicators were almost completely overlapping between the adenoma group and the serrated polyps group, and none of the differences reached statistical significance. This finding indicates that the two types of lesions may share a common background of risk factors in younger populations. However, as age increases, the proportion of adenomas continues to rise, while the proportion of serrated polyps shows a downward trend, presenting contrasting trajectories. The serrated pathway may be initiated and tends to reach “saturation” at a relatively young age, whereas the adenoma pathway remains continuously activated with the extension of metabolic exposure time. This age-dependent dynamic also explains why serrated polyps (227/412, 55.1%) outnumbered conventional adenomas (177/412, 43.0%) in our young cohort: the serrated-polyp group was dominated by hyperplastic polyps (282 lesions in 190 of 227 serrated-polyp cases, 83.7%)—particularly small (< 10 mm) and diminutive (≤ 5 mm) rectosigmoid lesions—which are common in young adults and only weakly age-dependent, whereas conventional adenomas are strongly age-dependent and remain uncommon at this age (ADR = 4.58%–13.23%). From the perspective of pathogenesis, it is speculated that the development of serrated polyps is more closely associated with gene mutations and epigenetic alterations, while the development of adenomas is more persistently driven by environmental and metabolic factors (14, 29).

Among patients with colonic adenomas, there were no significant differences in BMI, blood glucose, blood lipid levels, and ALT levels between adenomas located in the left colon and those in the right colon, and metabolic factors did not show a driving effect on the anatomical localization of adenomas. Among patients with serrated polyps, each 1 kg/m^2^ increase in BMI was associated with a 13% increase in the odds of the lesion being in the left colon (OR = 1.13, *p* = 0.036), while indicators such as fasting blood glucose and triglycerides did not exhibit a similar trend, suggesting that this effect of BMI may be independent of the classical metabolic pathway (30, 31).

In this study, the distribution of polyps in the left and right colon was generally balanced across the three groups, with no obvious left- or right-side predominance. This differs from the common left-colon-predominant distribution pattern observed in middle-aged and elderly populations (32, 33). Right-sided polyps are generally more closely associated with the serrated pathway (14, 33). Furthermore, this distribution characteristic indicates that for colonoscopy examinations performed on young populations, close observation of the entire colon should be emphasized, and the quality of observation during colonoscope withdrawal from the right colon should not be neglected.

This study found that among young adults aged 18–39 years, the gender gap in adenoma detection rate shows an expanding trend with increasing age, but the interaction effect between age and gender was only marginally significant (*p* = 0.059). In the 35–39-year age group, the adenoma detection rate in men was 2.24 times that in women (17.92% vs. 8.00%). In the gender-stratified analysis, the increase in adenoma risk with age among men (odds ratio per year, OR = 1.13) is significantly larger than that among women (OR = 1.05 per year), and only the trend for men reached statistical significance. The interaction effect does not meet the conventional significance threshold (*p* < 0.05), which may be associated with the relatively small absolute number of adenoma cases in women. These gender-stratified and age-by-gender interaction analyses are exploratory and hypothesis-generating; the subgroup estimates therefore warrant cautious interpretation. From the perspective of public health, the adenoma detection rate among men over 35 years old has reached a relatively high level, with a significant gap compared with that among women. This indicates that age and gender should be considered jointly when formulating colorectal cancer screening strategies for young populations. For men over 35 years old presenting with gastrointestinal symptoms, the benefits of colonoscopy should be evaluated.

Based on the analysis, this study puts forward the following clinical considerations: First, young men over 35 years old should be regarded as a key population for colorectal cancer screening. When patients present with gastrointestinal-related clinical symptoms, the benefits of colonoscopy screening should be evaluated. Second, screening results for fatty liver disease and metabolic syndrome can serve as an auxiliary reference for colonoscopy decision-making among young populations—the risk of colorectal neoplasia in young patients with concurrent fatty liver disease or metabolic abnormalities may be underestimated. Third, the proportion of serrated polyps is relatively high among young populations; thus, endoscopists should improve their ability to identify flat-type and right-sided serrated polyps to avoid missed diagnoses.

These findings should be interpreted as a cross-sectional snapshot rather than a temporal trend. The present cohort was ascertained over a single, recent period (2023–2024) at one center; therefore, the age-stratified detection rates represent point prevalence within that window and cannot disentangle a true increase in underlying lesion incidence from period and birth-cohort effects or from changes in diagnostic intensity. This distinction is critical for colonoscopy-based cohorts: rising detection among young adults can reflect greater diagnostic activity—wider use of colonoscopy and fecal immunochemical testing and heightened pathologist awareness—at least as much as a biological increase in disease, as recent population-based data demonstrating rising adenoma detection yet stable invasive colorectal cancer incidence and mortality have shown (34). The age gradient we report therefore describes how detection varies with age within this cohort and should not be read as population-level incidence or as direct evidence for earlier screening. As early-onset colorectal cancer continues to rise in successively younger birth cohorts, these contemporary point-prevalence estimates may become outdated rapidly and should be extrapolated to other periods, settings, or populations only with caution; defining the true temporal trajectory will require population-based, indication-complete longitudinal surveillance.

This study has certain limitations. First, as a single-center retrospective study with the proportion of asymptomatic individuals recruited from health examinations being less than 30%, selection bias is present, which may lead to an overestimation of the adenoma detection rate. Therefore, caution should be exercised when extrapolating the study results to the general asymptomatic population. Second, differences in the recruitment time window across different age groups resulting from sample size accumulation requirements may introduce time-varying confounding factors, although confining enrollment to a single recent period (2023–2024) is, for a cross-sectional prevalence study, preferable to a longer heterogeneous window in minimizing secular changes in practice and pathology criteria. Third, the lack of refined data on diet, physical activity, tobacco and alcohol consumption limits the exploration of the underlying mechanisms linking metabolic abnormalities and adenoma development. In addition, gender imbalance in sample size resulting from the low adenoma detection rate among young women may have reduced the statistical power for subgroup interaction analysis. Colorectal cancer is intrinsically uncommon in adults aged 18–39 years, and powering each histology-, age-, and gender-stratified cell to 80% would demand a sample size that is impractical for a single-center study; the present cohort was therefore powered for its primary objective, with all stratified and interaction analyses regarded as exploratory. Definitive confirmation of these subgroup and interaction findings will require multicenter prospective studies. Future prospective cohort studies based on asymptomatic populations, combined with lifestyle questionnaires and biomarker assays, are required to further validate the findings of this study.

## Conclusion

This study demonstrates a significant age-dependent increase in colorectal adenoma and advanced neoplasia detection rates among adults aged 18–39 years. Men, particularly those aged 35–39 years, constitute the highest-risk subgroup. Notably, the rising trajectories of adenoma detection paralleled increases in BMI, lipids, and fatty liver prevalence, implicating metabolic factors in early-onset colorectal neoplasia. While adenomas and serrated polyps exhibited similar clinical profiles, they displayed divergent age-related trends: adenomas increased while the proportion of serrated polyps decreased with age. Furthermore, elevated BMI was associated with left-colonic distribution in serrated polyps but not in adenomas, suggesting differential metabolic susceptibility between these pathological subtypes.

## Data Availability

The raw data supporting the conclusions of this article will be made available by the authors, without undue reservation.

## References

[1] Morgan E Arnold M Gini A Lorenzoni V Cabasag CJ Laversanne M . Global burden of colorectal cancer in 2020 and 2040: Incidence and mortality estimates from GLOBOCAN. Gut. (2023) 72:338–44. doi: 10.1136/gutjnl-2022-327736 36604116

[2] Diao X Guo C Jin Y Li B Gao X Du X . Cancer situation in China: An analysis based on the global epidemiological data released in 2024. Cancer Commun (London England). (2025) 45:178–97. doi: 10.1002/cac2.12627 39659114 PMC11833671

[3] Patel SG Karlitz JJ Yen T Lieu CH Boland CR . The rising tide of early-onset colorectal cancer: A comprehensive review of epidemiology, clinical features, biology, risk factors, prevention, and early detection. Lancet Gastroenterol Hepatol. (2022) 7:262–74. doi: 10.1016/S2468-1253(21)00426-X 35090605

[4] Du M Drew DA Goncalves MD Cao Y Chan AT . Early-onset colorectal cancer as an emerging disease of metabolic dysregulation. Nat Rev Endocrinol. (2025) 21:686–702. doi: 10.1038/s41574-025-01159-z 40781551 PMC12707992

[5] Lv W Wang S Duan L Wu Y Tian X Dong Z . Estimating the global macroeconomic impact of colorectal cancer: Evidence from Global Burden of Disease 2021 and Value of a Statistical Life Year framework. Int J Surg (London England). (2026) 112:9577–86. doi: 10.1097/JS9.0000000000004687 41664847 PMC13105516

[6] Spaander MCW Zauber AG Syngal S Blaser MJ Sung JJ You YN . Young-onset colorectal cancer. Nat Rev Dis Primers. (2023) 9:21. doi: 10.1038/s41572-023-00432-7 37105987 PMC10589420

[7] Rydbeck D Asplund D Bock D Haglind E Park J Rosenberg J . Younger age at onset of colorectal cancer is associated with increased patient's delay. Eur J Cancer (Oxford Engl 1990). (2021) 154:269–76. doi: 10.1016/j.ejca.2021.06.020 34298377

[8] Demb J Kolb JM Dounel J Fritz CDL Advani SM Cao Y . Red flag signs and symptoms for patients with early-onset colorectal cancer: A systematic review and meta-analysis. JAMA Netw Open. (2024) 7:e2413157. doi: 10.1001/jamanetworkopen.2024.13157 38787555 PMC11127127

[9] Patel SG May FP Anderson JC Burke CA Dominitz JA Gross SA . Updates on age to start and stop colorectal cancer screening: Recommendations from the U.S. Multi-Society Task Force on Colorectal Cancer. Am J Gastroenterol. (2022) 117:57–69. doi: 10.14309/ajg.0000000000001548 34962727

[10] Expert Group On Early Diagnosis And Treatment Of Cancer CSOO . Expert consensus on the early diagnosis and treatment of colorectal cancer in China (2023 edition). Zhonghua Yi Xue Za Zhi. (2023) 103:3896–908. 38129166 10.3760/cma.j.cn112137-20230804-00164

[11] He X Lv X Zhang B Ying X Hu C Zhou X . Adenoma detection rate in average-risk population: An observational consecutive retrospective study. Cancer Control J Moffitt Cancer Center. (2023) 30:1379356005. 37528552 10.1177/10732748231193243 PMC10399273

[12] Shen YQ Di MY Deng YF Xu MQ Xue ZY . Profile of colorectal polyps in young patients: A retrospective study. Medicine. (2025) 104:e42475. doi: 10.1097/MD.0000000000042475 40388751 PMC12091607

[13] Dominitz JA Ladabaum U Holub JL Issaka RB Ko CW Robertson DJ . Association between adenoma detection rate and prevalent colorectal cancer detection rate in a national colonoscopy registry. Gastroenterology. (2025) 169:1489–98. doi: 10.1053/j.gastro.2025.06.009 40523534

[14] Aiderus A Barker N Tergaonkar V . Serrated colorectal cancer: Preclinical models and molecular pathways. Trends Cancer. (2024) 10:76–91. doi: 10.1016/j.trecan.2023.09.011 37880007

[15] Crockett SD Nagtegaal ID . Terminology, molecular features, epidemiology, and management of serrated colorectal neoplasia. Gastroenterology. (2019) 157:949–66. doi: 10.1053/j.gastro.2019.06.041 31323292

[16] Zessner-Spitzenberg J Waldmann E Rockenbauer L Demschik A Penz D Trauner M . Polyp size is associated with colorectal cancer death across histologic polyp subtypes: A retrospective study of a screening colonoscopy registry. Endoscopy. (2024) 56:820–7. doi: 10.1055/a-2339-0146 38936414

[17] Sadowski DC Michael R Kolber GA Hickle L Hilsden R McLean DR . Post-polypectomy surveillance: Follow-up recommendations from the Alberta Colorectal Cancer Screening Program. J Can Assoc Gastroenterol. (2024) 7(4):319–28. doi: 10.1093/jcag/gwae007 39139219 PMC11317626

[18] Chen Y Padilla Aponte J Wang K Du M Lu Y Polychronidis G . Comparative risk of high-risk neoplasia after polypectomy among individuals aged below 50 years versus 50 years and older. Gut. (2025) 75:72–80. doi: 10.1136/gutjnl-2025-335275 40850745

[19] Gong Y Zheng Y Wu R Liu M Li H Zeng Q . Detection rates of adenomas, advanced adenomas, and colorectal cancers among the opportunistic colonoscopy screening population: A single-center, retrospective study. Chin Med J-Peking. (2023) 136:159–66. doi: 10.1097/CM9.0000000000002435 36692899 PMC10106243

[20] Yeh J Lin C Hsiao P Perng D Chen J Hung K . Prevalence and predictive factors of colorectal sessile serrated lesions in younger individuals. Endoscopy. (2024) 56:494–502. doi: 10.1055/a-2272-1911 38378019 PMC11583004

[21] Kumbhari V Behary J Hui JM . Prevalence of adenomas and sessile serrated adenomas in Chinese compared with Caucasians. J Gastroen Hepatol. (2013) 28:608–12. doi: 10.1111/jgh.12100 23278321

[22] Qiu Y Fu X Zhang W Xu Y Xiao L Chen X . Prevalence and molecular characterisation of the sessile serrated adenoma in a subset of the Chinese population. J Clin Pathol. (2014) 67:491–8. doi: 10.1136/jclinpath-2013-202092 24570042

[23] Waldmann E Jiricka L Zessner-Spitzenberg J Majcher B Rockenbauer L Penz D . Differences between men and women with respect to colorectal cancer mortality despite screening colonoscopy. Gastrointest Endosc. (2024) 99:998–1005. doi: 10.1016/j.gie.2024.01.001 38184115

[24] Wang L Tu Y Chen L Zhang Y Pan X Yang S . Male-biased gut microbiome and metabolites aggravate colorectal cancer development. Advanced Sci (Weinheim Baden-Wurttemberg Germany). (2023) 10:e2206238. doi: 10.1002/advs.202206238 37400423 PMC10477899

[25] Wu Z Huang Y Zhang R Zheng C You F Wang M . Sex differences in colorectal cancer: With a focus on sex hormone-gut microbiome axis. Cell Communication Signaling CCS. (2024) 22:167. doi: 10.1186/s12964-024-01549-2 38454453 PMC10921775

[26] Zhou X Sevilla-Gonzalez M Phipps AI Udler M Castellví-Bel S Chan AT . Diabetogenic processes for insulin resistance-linked hyperinsulinaemia are associated with colorectal cancer. Diabetologia. (2026) 69:942–52. doi: 10.1007/s00125-025-06631-z 41398395 PMC12957015

[27] Wu C Targher G Byrne CD Younossi ZM Armstrong MJ Luu HN . Colorectal cancer in metabolic dysfunction-associated steatotic liver disease: An international Delphi consensus statement. Gut. (2026) 75(10):2009–18. doi: 10.1136/gutjnl-2025-337374 41770836

[28] Li C Cai C Wang C Chen X Zhang B Huang Z . Gut microbiota-mediated gut-liver axis: A breakthrough point for understanding and treating liver cancer. Clin Mol Hepatol. (2025) 31:350–81. doi: 10.3350/cmh.2024.0857 39659059 PMC12016628

[29] Xin Y Liu C Cui J Wang Y Wu H . Lifestyle can exert a significant impact on the development of metabolic comorbidities in early-stage colorectal cancer patients. Front Nutr. (2025) 12:1551526. doi: 10.3389/fnut.2025.1551526 40686812 PMC12272228

[30] Bardou M Rouland A Martel M Loffroy R Barkun AN Chapelle N . Review article: Obesity and colorectal cancer. Aliment Pharm Ther. (2022) 56:407–18. doi: 10.1111/apt.17045 35707910

[31] Ungvari Z Fekete M Varga P Lehoczki A Fekete JT Ungvari A . Overweight and obesity significantly increase colorectal cancer risk: A meta-analysis of 66 studies revealing a 25-57% elevation in risk. Geroscience. (2025) 47:3343–64. doi: 10.1007/s11357-024-01375-x PMC12181496 39379738

[32] Javidi DJ Butterly LF Amos CI Mackenzie TA Robinson CM Anderson JC . Risk factors of proximal and distal advanced colorectal conventional and serrated neoplasia in adults younger than 50 years of age. Am J Gastroenterol. (2026), 10–14309. doi: 10.14309/ajg.0000000000003950 41677132 PMC13614758

[33] Sung H Siegel RL Laversanne M Jiang C Morgan E Zahwe M . Colorectal cancer incidence trends in younger versus older adults: An analysis of population-based cancer registry data. Lancet Oncol. (2025) 26:51–63. doi: 10.1016/S1470-2045(24)00600-4 39674189 PMC11695264

[34] Janssens S Van Herck K Francart J Van Gool B Audibert A Clinckaert C . Evaluating incidence and mortality trends of early-onset colorectal cancer and adenoma in Belgium. ESMO Gastrointestinal Oncol. (2026) 12:100340. doi: 10.1016/j.esmogo.2026.100340 42256517 PMC13234709

